# The Schisto Track: A System for Gathering and Monitoring Epidemiological Surveys by Connecting Geographical Information Systems in Real Time

**DOI:** 10.2196/mhealth.2859

**Published:** 2014-03-10

**Authors:** Onicio B Leal Neto, Cesar M Albuquerque, Jones O Albuquerque, Constança S Barbosa

**Affiliations:** ^1^Aggeu Magalhaes Research CenterSchistosomiasis Reference ServiceOswaldo Cruz FoundationRecifeBrazil; ^2^PPGIADepartment of Statistics and InformaticsFederal Rural University of PernambucoRecifeBrazil

**Keywords:** epidemiological survey, schistosomiasis, public health

## Abstract

**Background:**

Using the Android platform as a notification instrument for diseases and disorders forms a new alternative for computerization of epidemiological studies.

**Objective:**

The objective of our study was to construct a tool for gathering epidemiological data on schistosomiasis using the Android platform.

**Methods:**

The developed application (app), named the Schisto Track, is a tool for data capture and analysis that was designed to meet the needs of a traditional epidemiological survey. An initial version of the app was finished and tested in both real situations and simulations for epidemiological surveys.

**Results:**

The app proved to be a tool capable of automation of activities, with data organization and standardization, easy data recovery (to enable interfacing with other systems), and totally modular architecture.

**Conclusions:**

The proposed Schisto Track is in line with worldwide trends toward use of smartphones with the Android platform for modeling epidemiological scenarios.

## Introduction

### New Tools for Public Health Studies

Among the traditional aspects of the structures of public health studies, information is a fundamental element in planning and carrying out the actions. With the worldwide movement toward new technologies, new tools are being incorporated within public health studies in order to make information more dynamic and increasingly accessible to health care system users [1].

The growth of the Internet has popularized information within health care as mobile systems like smartphones have become more accessible among the population [2]. Mobile phones with remote access to the Internet also have other functional features; they can receive signals from the global positioning system, store files and data in flash memory, and manipulate text, among other functions. With the evolution of open source development platforms for smartphones, users with knowledge of software programming can develop specific applications for these devices and make them available on the Internet for download. Using these applications for health care services has the great advantage of direct contact between the administration and the population, taking these parties to be participants in the processes at the locations where the manifestations of health and illness develop.

### The Android Platform

The Android platform (the operating system for smartphones developed by Google) has provided a new alternative for computerizing epidemiological studies, through using this, the data gathering process in the field is facilitated, errors are reduced, instantaneous communication is enabled, and a virtual database can be stored on the Internet [2]. The advances among operating systems, computing platforms, programming languages, and development frameworks are becoming integrated and being reapplied to mobile devices. This is improving the way in which epidemiological investigations are conducted, thereby supplying scenarios and responses for resolving public health problems [3]. The Epi Schisto Risk Modeling research group has been working on the development platform called “A New Kind of Simulator”, in which a variety of computing tools form instruments for routine use in public services that deal with epidemiological and environmental surveillance. In this regard, one of the products conceived for exploitation of these services was developed with the aim of gathering and transmitting epidemiological data on schistosomiasis in real time, using the Android platform, with a view to future use of electronic tools to optimize the routine of health surveillance sectors.

### Schistosomiasis as a Case Study for the Instruments

Schistosomiasis was chosen as a backdrop and case study on using and validating these instruments since it has considerable epidemiological representation in relation to diseases occurring in different regions of Brazil, and its control and elimination is a challenge. Snails of the genus *Biomphalaria* transmit this disease, and its etiological agent is the parasite *Schistosoma mansoni* [4]. In Pernambuco, this disease is expanding to coastal areas used for vacations and tourism. Several studies on these areas have diagnosed human cases and new concentrations of the vector mollusks of the disease [5,6], and have shown that there is a need for investments in tools for rapid and precise epidemiological diagnosis that might prevent or minimize outbreaks of acute cases.

### Aim of the Study

The aim of the present study was to construct and present a tool to be used by field workers for use in epidemiological surveys in order to collect and transmit data in real time. In this manner, emerging technologies and their application to public health would become aligned and greater security and speed in consolidating and storing the data would be promoted. The “schistosomiasis model” was used to construct this tool, and its system was fed with all the variables (biological, environmental, and operational) used in epidemiological surveys that would be related to the parasite, vector mollusk, and human cases. This study was conducted through a partnership between the Department of Information Technology of the Federal Rural University of Pernambuco and the Schistosomiasis Reference Services and Laboratory of the Aggeu Magalhães Research Center, Oswaldo Cruz Foundation.

## Methods

### The Schisto Track Application

The Schisto Track application is a data capture and analysis tool comprising a combination of a mobile application and a server. Its construction was designed to meet the needs of a traditional epidemiological survey. Hence, it was planned in four segments: (1) registration of homes/individuals, (2) registration of breeding sites, (3) consultation of registered data, and (4) registration of paths followed. The first two of these segments used the *SQLite* database model. This database was fed with variables that had been validated in other epidemiological surveys that had aimed to identify foci of vector mollusks, diagnose human cases, and spatially locate information relating to schistosomiasis occurrences [5-8]. For each set of variables, information fields were set up (Tables 1 and 2).

**Table 1:** Variables to be gathered for registering breeding sites and foci of vector mollusks.

Field of information	Description	Epidemiological relevance
Photography	Direct observation and visual storage of macro-environmental elements.	Evaluation of the vegetation type, substrate, water surface dimensions, and proximity to homes and sewage ditches.
Number of snails collected	Quantification of the number of mollusk specimens collected.	Analysis on the snail population density per breeding site.
Collection station number	Coded register of each component station of the breeding site.	Reference for mapping each breeding site and systematizing collections.
Location	Recording of a pair of coordinates for each breeding site.	Composing of georeferenced points in the geographical information system, available on the Internet.
Observation	Open field for recording additional information on the location and completing the address.	Social representation of the area through localization recognized by people living nearby.
Breeding site classification	Permanent or temporary	Identification of whether the breeding site structure allows flooding or continual water collection, or whether it is of limited nature regarding its permanence in the environment (for example, only existing during rainy periods).
Type of breeding site	Ditch/canal; stream/small river; marsh/bog; lake/river	Identification of the type of snail habitat.
Water level	Deep; medium; low; dry	Viability of mobility and permanence of snails in the breeding sites.
Salinity level	0 to 0.4; 0.5 to 0.8; > 0.8	Snail resistance in habitats of low, medium, and high salinity.
pH	< 7; = 7; > 7	Snail resistance in acidic or basic environments.

**Table 2:** Variables to be gathered for registering homes and individuals participating in the study.

Field of information	Description	Epidemiological relevance
Municipality	Identification for simultaneous surveys in several municipalities.	Study territory
Locality	Identification of official and unofficial geographical territories.	Identification of areas covered by the Family Health Strategy, Community Health Agent Program, or Endemic Disease Agent Program.
Home number	Specific identification for each home participating.	Registration for mapping the area.
Number of receptacles handed out	Quantity of collecting receptacles handed out to participants.	Analysis to estimate prevalences and sample losses.
Name	Identification of study participants by name.	Preparation of parasitological reports.
Date of birth	Identification of participants’ ages.	Data for analyses according to age group, descriptive statistics, and other analysis models.
Sex	Male or female	Data for analysis according to sex, descriptive statistics, and other analysis models.
Sample number	Registration of the epidemiological sample to be analyzed.	Coding in the database.

### The Structure of the System

The structure of the system was divided into two modules: (1) the application (app), and (2) the server, thus making it possible to add and extract functional features in accordance with the needs imposed by the study in question. For this to be developed, the Android software development kit had to be used, and the Java language was used with a view to optimizing the results. This language was used because of its properties of robustness, security, distribution, portability, neutral architecture, and interpretability, while also presenting high performance [9,10].

The app was constructed in the Eclipse environment (Helios version), by means of the Android development tools (ADT) plugin, in order to facilitate production, tests, and project compilation. By using the ADT, it was possible to perform Android emulation directly from Eclipse, making use of all its resources, such as debug. It was also possible to control the emulator, view logs, and simulate sending text messages, or to make telephone calls, in addition to the capacity to view and send files, run the garbage collector, and view the heap memory, along with other possibilities intrinsic to the functioning of the Android platform in the device [11].

As the data were registered, they were instantaneously transferred through the Internet by means of a third generation (3G) or wireless network to the Web server, where the information would form the MySQL database, with the option of generating spreadsheets in the Microsoft Excel format for use in spatial analysis programs by means of ArcGIS (Environmental Systems Research Institute).

The Schisto Track framework enabled interfacing with other systems and had totally modular architecture. It was integrated with the Web mapping service app Google Maps and a three dimensional (3D) cartographic platform, and the app programming interface (API) was available for all the sites that could be accessed, free of charge, by users and developers, thereby facilitating viewing of the spatially distributed data [12].

For remote data reception, the Web environment server was divided into a front end or presentation layer, and a back end or administrator panel. Both of these were developed using Hypertext Markup Language 4.01 and Ajax. To view the trails that were generated from the paths followed by the technicians, Google Maps JavaScript API V3 was used.

## Results

### The Schisto Track Application

**Figure 1:**
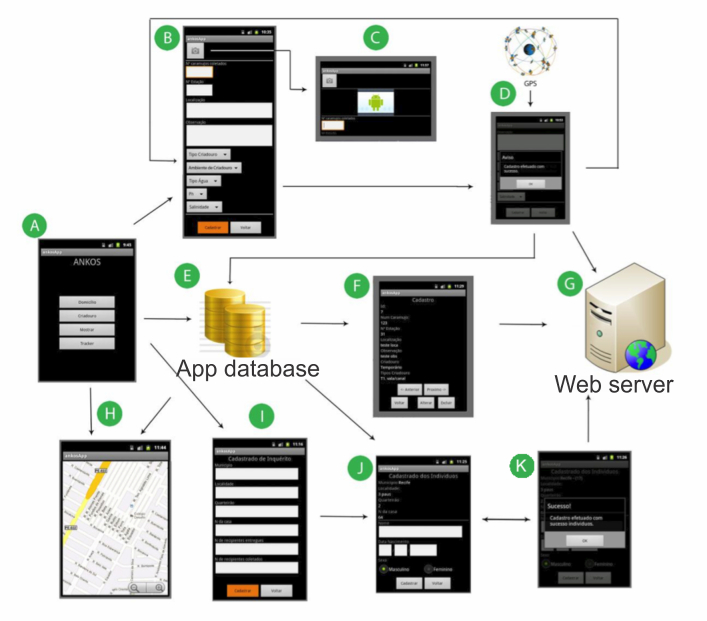
Initial version of the Schisto Track app. Global positioning system (GPS); Application (App); A New Kind of Simulator (ANKOS).

The Schisto Track was shown to be a tool capable of automating the activities of registering breeding sites and homes, organizing and standardizing the data, and facilitating information recovery. It enabled interfacing with other systems and had totally modular architecture. It was integrated with Google Maps, and the API was available for all the sites that could be accessed, free of charge, by users and developers, thereby facilitating viewing of the spatially distributed data [12]. An initial version of the Schisto Track app was developed and tested between March and December 2011, using real situations and simulations for epidemiological surveys on schistosomiasis. Figure 1 shows the workflow.

### Web Server

**Figure 2:**
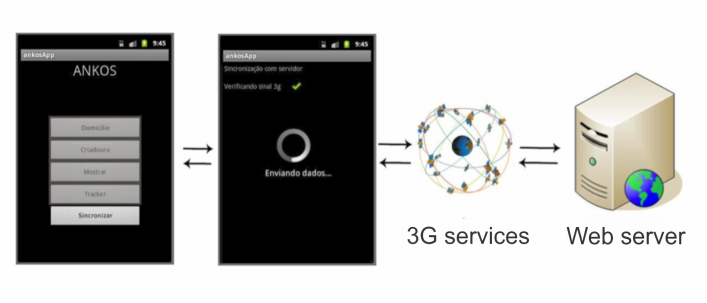
Synchronization of the Schisto Track with the Web server. A New Kind of Simulator (ANKOS); application (App).

To view the information registered in the field that was sent remotely to the Web server, two access levels were created: (1) open access for ordinary users, and (2) restricted access for project researchers. The information made available through open access related to the spatial distribution of the breeding site points that had been demarcated, which were viewed on the 3D cartographic platform of Google Earth. Restricted access, obtained through a registration module with identification using a log-in and password, enabled consultation and editing of the database containing all of the information from the study, and also allowed exporting of files to specific formats that could be used in geostatistical analysis software. The Schisto Track was designed for use by several users and in several locations at the same time. The different access levels ensured that the information stored in the database remained secure and trustworthy, thus avoiding inclusion of false information or access by unregistered individuals. This information was stored in databases located inside the device (SQLite) and, when a 3G mobile network or equivalent (EDGE, HSPDA) was present, the data were synchronized and sent to the Web server (Figure 2 shows this synchronization).

### Registration of Mobiles and Field Technicians

To ensure data identification and security, the functional features of the system required registration of the smartphones by means of the standard number of each device on the Android platform (identification-ID and serial). In addition, the ID of each field technician who was going to conduct the epidemiological survey was linked to the work tool. Thus, it was possible to make an association with the person responsible for the data collection.

From the initial screen of the Schisto Track, one can see that the data gathered in the field were transmitted and could be viewed in real time by any individual who wished to access the electronic address of the study. This artifice promoted dissemination of information warning about the areas at risk that had been identified, thereby ensuring that the communities were empowered and mobilized to take the individual and collective preventive measures that might be needed (see Multimedia Appendix 1).

## Discussion

### Operational Stages of Data Gathering

The operational stages for data gathering in epidemiological surveys constitute an arduous and onerous process involving a large amount of manpower for manually filling in record cards and data sheets that are later on transcribed in order to enter the data into computerized spreadsheets. This is a tiresome practice that is liable to involve transcription errors and information bias. An epidemiological survey conducted in the coastal locality of Porto de Galinhas, in the municipality of Ipojuca [6], registered 5800 participants and, for this, it was necessary to maintain a workforce of 10 data gatherers in the field for 11 months. This demonstrates that managing the process of field data acquisition, consolidation, and analysis is an exhausting challenge. In light of situations like this, the Schisto Track app constitutes a valuable tool since it imprints dynamism, speed, and precision on the data gathered, thereby avoiding transcription errors and bringing greater benefits to population based studies, in terms of security and logistics.

### The Schisto Track Proposal

The Schisto Track proposal is in line with the worldwide trend toward using smartphones with the Android platform for modeling epidemiological scenarios [1,13,14]. Moreover, use of mobile phones has already been highlighted as a solution for transmitting health care information from remote areas, and as a tool for managing logistic processes, as well as serving as a tool for producing crowdsourcing and other health care activities [2,15-19].

The Google Maps platform, which is included in the app, provides immediate spatial positioning of the events, the vector foci that are detected, and the human cases that are diagnosed. The use of this platform as the base map for epidemiological and environmental studies is considered to be a modern trend. This platform has also been used by researchers around the world who have done so from the perspective of constructing dynamic epidemiological scenarios [20-22]. The information technology used in epidemiology and surveillance of health problems has evolved from concepts such as “Infodemiology” and “Infoveillance”. In addition to the integration with electronic media [23,24] that is represented by open platform mobile devices, the Internet, and social networks, information technology promotes the dissemination of information by the health care system, service agents, and users because of the speed and quality of the information generated. These advances in health care information technology are in line with the proposal for participative epidemiology, in which it is recommended that indicators and situations should be made available on social networks, so that these can be explored by health care sector administrators and users. In this manner, a set of new tools and trends for coping with public health problems is enabled [25].

In Brazil, few tools for this purpose are available, and none of them present the robust characteristics that are as suitable for health care services as the Schisto Track proposal. This app brings together the practicality of a low cost tool that is easy to use with the importance of a public health instrument that aims to improve the processes of data capture and spatial environmental diagnosis in epidemiological surveys. For epidemiology, this is an instrument that is also applicable to other disease models, thus constituting an alternative for improving routine practice in health care and health surveillance services.

## Multimedia Appendix 1

Screenshot from the ANKOS website, showing the possibility of making real time consultations for each breeding.

## Abbreviations

3D: three dimensional
3G: third generation
ADT: android development tools
ANKOS: A New Kind of Simulator
API: app programming interface
app: the application
ID: identification
GPS: global positioning system
