# Supplementary figures and images for: The Schisto Track: A System for Gathering and Monitoring Epidemiological Surveys by Connecting Geographical Information Systems in Real Time
Source: JMIR Mhealth Uhealth. 2014 Mar 10;2(1):e10. doi: 10.2196/mhealth.2859 (PMC4114433; doi:10.2196/mhealth.2859)

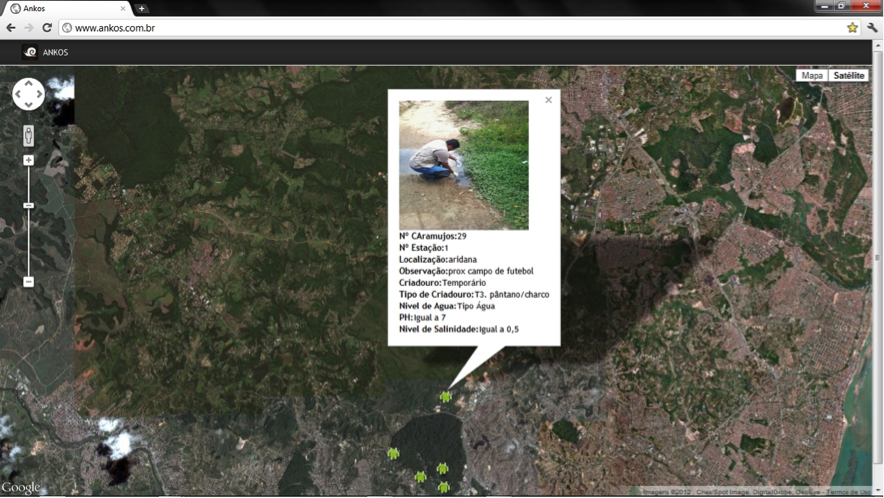

Supplement: Supplementary file 1 [file mhealth_v2i1e10_app1.png]
